# Role of RNA methyltransferases in tissue renewal and pathology

**DOI:** 10.1016/j.ceb.2014.06.006

**Published:** 2014-12

**Authors:** Sandra Blanco, Michaela Frye

**Affiliations:** Wellcome Trust – Medical Research Council Cambridge Stem Cell Institute, University of Cambridge, Tennis Court Road, Cambridge CB2 1QR, United Kingdom

## Abstract

Over the last five decades more than 100 types of RNA modifications have been identified in organism of all kingdoms of life, yet their function and biological relevance remain largely elusive. The recent development of transcriptome-wide techniques to detect RNA modifications such as N^6^-methyladenosine (m^6^A) and 5-methylcytidine (m^5^C) has not only created a new field of research ‘*the epitranscriptome*’ but also featured essential regulatory roles of RNA methylation in a wide range of fundamental cellular processes. Here, we discuss the current knowledge of m^6^A and m^5^C RNA methylation pathways and summarize how they impact normal tissues and contribute to human disease.

**Current Opinion in Cell Biology** 2014, **31**:1–7

This review comes from a themed issue on **Cell cycle, differentiation and disease**

Edited by **Stefano Piccolo** and **Eduard Batlle**

For a complete overview see the Issue and the Editorial

Available online 10th July 2014

**http://dx.doi.org/10.1016/j.ceb.2014.06.006**

0955-0674/© 2014 The Authors. Published by Elsevier Ltd. This is an open access article under the CC BY license (http://creativecommons.org/licenses/by/3.0/).

## Introduction

Post-transcriptional regulation of gene expression ultimately determines the rate of protein translation and is therefore crucial for virtually all cellular processes. Post-transcriptional modifications add complexity to RNA-mediated functions by regulating how and when a primary RNA transcript is converted into mature RNA. There are around 150 known RNA modifications [1], yet our knowledge about their occurrence and function in RNA is still very limited. The existence of methylated bases in RNA including C^5^-methylcytidine (m^5^C) and N^6^-methyladenosine (m^6^A) has been described 50 years ago [2]. However, until only very recently, m^5^C for instance was thought to be mainly restricted to the stable and highly abundant transfer RNAs (tRNAs) and ribosome RNAs (rRNAs) [3].

The recent development of novel transcriptome-wide approaches to capture global m^5^C and m^6^A RNA methylomes has not only restored scientific interest in the field but also contributed to a better understanding how gene expression is regulated at different levels. In only a couple of years it became evident that post-transcriptional methylation of both cytosines and adenosines regulate fundamental cellular processes that are essential for normal development. The importance of a tightly controlled deposition of both m^5^C and m^6^A into RNA is further underscored by the strong link of loss-of-function mutations in methylating and demethylating enzymes to several severe human diseases.

## Post-transcriptional 5-methylcytidine

**Figure 1:**
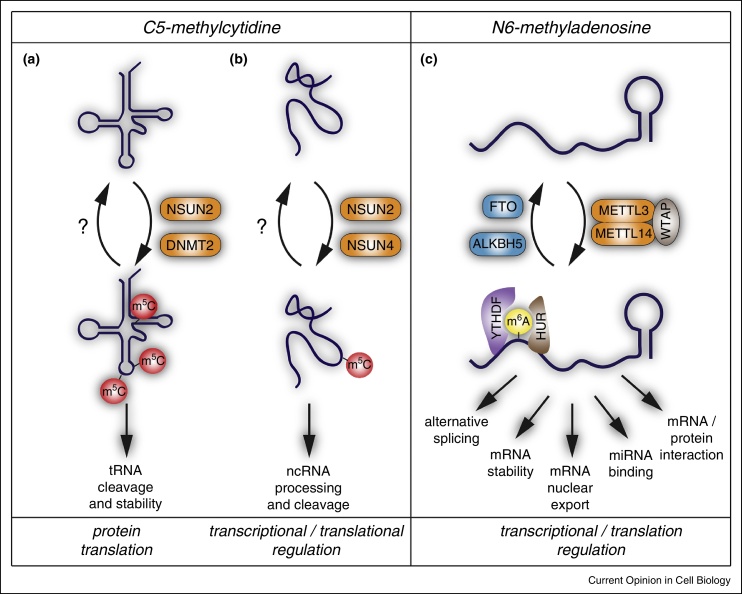
Regulation and function of RNA methylation. C^5^-methylcytidine (m^5^C) is a common modification in **(a)** tRNAs and **(b)** other non-coding RNAs (ncRNAs). NSun2, NSun4 and Dnmt2 can catalyze methylation of cytosine-5 but no m^5^C-demethylases have been reported yet (a and b). **(c)** N6-methyladenosine (m^6^A) is an abundant internal modification in mRNA. Its deposition is dynamically regulated by methylases (Mettl3 and Mettl14) and demethylases (Fto and AlkBH5). Accurate and adequate methylation levels dictate the fate, processing, interaction with ‘readers’ (YTHDF, HUR) and further function of methylated RNAs. All reported molecular functions relate to the regulation of transcriptional and translational processes.

Over the last years, several methods have been developed to globally detect 5-methylcytidine in RNA. Bisulfite sequencing was first adapted for detecting m^5^C in RNA and confirmed that m^5^C can be reproducibly and quantitatively detected in tRNA and rRNA (Figure 1a and b) [4]. RNA bisulfite conversion in combination with next generation sequencing further identified m^5^C in both coding and non-coding RNAs in addition to tRNAs and rRNAs [5, 6•]. One limitation of RNA bisulfite sequencing is that ideally the data need to be compared to cells lacking the specific RNA methyltransferases to confirm the signals. Indeed, only a small fraction of methylated RNAs identified by bisulfite sequencing overlapped with the specific RNA targets of the cytosine-5 RNA methylases Dnmt2 and NSun2 [3].

Two recently developed methods based on RNA immunoprecipitation approaches followed by next generation sequencing identified Dnmt2- and NSun2-specific RNA methylation targets [7••, 8••]. In spite of all system-wide approaches, Dnmt2-mediated methylation seems to be restricted to only three tRNAs: Gly^GCC^, Asp^GTC^ and Val^AAC^ [8••, 9, 10]. The vast majority of NSun2-mediated methylation was found in a wide range of tRNAs, but in addition NSun2 also targeted other non-coding and a small number of coding RNAs [7••, 8••]. Among the non-coding RNAs, NSun2 consistently methylated vault RNAs [7^••^]. Hypomethylation of vault RNA at NSun2-mediated sites altered its processing patterns into small microRNA like molecules that can bind to Argonautes and regulate mRNAs [7^••^].

NSun2-mediated methylation of mRNAs remains enigmatic. Synthetic cytosine-5 methylated mRNAs can be more stable and loss of NSun2-mediated methylation in the 3′UTR of p16 has been reported to reduce its stability [11]. Yet we have shown recently that virtually none of the mRNAs potentially methylated by NSun2 changed in abundance in NSun2 depleted cells [7^••^].

## Biological roles of cytosine-5 RNA methylases

RNA m^5^C methyltransferase belong to a large and highly conserved group of proteins, yet their RNA substrate specificity is predicted to be different [12]. Pioneering work in single cell organisms shed light on the enzymatic formation as well as the molecular and biological functions of m^5^C in RNA and is reviewed elsewhere. For space reasons, we will focus on the biological roles of m^5^C methyltransferases in multicellular organisms.

## The DNA methyltransferase homolog Dnmt2

Among all RNA methyltransferases Dnmt2 is the best studied, yet mostly for its potential function in methylating DNA. Dnmt2 shares almost all sequence and structural features of DNA methyltransferases [13]. However, over the last years it became evident that Dnmt2 plays no major role in influencing global DNA methylation. Dnmt2-deficient mouse embryonic stem (ES) cells do not display altered genomic methylation patterns and organisms expressing only Dnmt2 as the sole candidate DNA methyltransferase gene lack genomic methylation patterns [14, 15].

Dnmt2 was one of the first cytosine-5 RNA methylases identified in a multicellular organism [16^••^]. Although Dnmt2-mediated methylation of cytosine 38 in the anticodon loop of tRNA^Asp^ was conserved in plant, flies and mice, none of these organisms lacking the functional Dnmt2 protein displayed any morphological differences to their wild-type counterparts [16^••^]. In contrast, morpholino-mediated loss of Dnmt2 in zebrafish reduced the size of the morphants by half and specifically affected liver, retina and brain development due to a failure to conduct late differentiation [17]. Over-expression of Dnmt2 on the other hand prolonged the life span of *Drosophila* by more than 50% and increased the resistance to stress [18]. In line with these studies, *Drosophila* Dnmt2 loss-of-function mutants showed reduced viability under stress conditions, and Dnmt2-mediated methylation protected tRNAs from stress-induced ribonuclease cleavage (Figure 1a) [9].

Cleavage of tRNAs is a conserved response to several stress stimuli in eukaryotes and the tRNA fragments are produced to repress translation by displacing translation initiation and elongation factors from mRNAs or by interfering with efficient transpeptidation [19, 20, 21]. However, whether and how increased tRNA cleavage in *Drosophila* Dnmt2 mutants is directly linked to stress tolerance and protein translation is currently unknown. While tRNA cleavage is mediated by angiogenin in mammals, the only identified tRNA nuclease in *Drosophila* so far is Dicer [22]. Interestingly, also expression of Dicer is down-regulated by oxidative stress and Dicer knockout cells can be hypersensitive towards oxidative stress whereas its over-expression confers stress resistance [23]. Other functions that have been linked to Dnmt2 but may be independent of its tRNA methyltransferase activity are silencing of retro-transposons and control of RNA viruses in *Drosophila* as well as RNA-mediated paramutations in the mouse [24]. Together, these data implicate that Dnmt2 is functionally redundant for normal development of most multicellular organisms but implicated in cellular stress responses at least in adult flies [24].

## The NOP2/Sun (NSun) RNA methyltransferase family

At least two more enzymes NSun2 and NSun4 can generate 5-methylcytidine in RNA in mammals (Figure 1a and b) [25, 26]. Both belong to the S-Adenosylmethionine (AdoMet)-dependent methyltransferase superfamily and at least five more putative m^5^C RNA methylases in mammals (NOP2, NSun3, and NSun5–7) are predicted to methylate RNA based on sequence conservation of key catalytic residues [12]. Although the substrate specificities are unknown, NSun1 and NSun5, in addition to NSun2 and Nsun4, have been identified as mRNA-binding proteins [27]. The biological functions of most members of the NSun-protein family is largely unknown, although all of them are expressed during mouse embryogenesis and NSun2–7 are all enriched in the developing brain [28].

NSun2 was first described in the mammalian epidermis as a transcriptional target of the proto-oncogene c-Myc [25]. NSun2 is up-regulated in a wide range of cancers and knockdown of NSun2 in human squamous-cell-carcinoma xenografts decreased their growth [25, 29]. NSun2 is a nucleolar protein that is regulated by Aurora B kinase and promotes cell division by stabilizing the mitotic spindle in cancer cell lines, yet this function seems independent of its methyltransferase activity and has yet to be confirmed *in vivo* [30, 31].

Interestingly, deletion of NSun2 in mice caused a phenotype resembling deletion of Dnmt2 in zebrafish. NSun2 knockout mice are smaller than their littermates and late differentiation is delayed or blocked in specific tissues including skin and testis [32, 33]. In humans, several genetic mutations in the NSUN2 gene have been identified and primarily cause autosomal-recessive intellectual disability and a Dubowitz-like syndrome [34, 35, 36•]. The common symptoms of the disorder include growth and mental retardation, unusual faces, and cutaneous abnormalities [34, 35, 36•]. Whether and how loss of RNA methylation is the underlying cause of all the symptoms of these complex diseases is currently unknown. However, similar to the human syndrome, deletion of the NSun2 ortholog in *Drosophila* caused severe short-term-memory deficits [35]; and simultaneous deletion of Dnmt2 and NSun2, which abrogates all tRNA methylation, specifically affected brain, liver, and adipose tissue development due to impaired differentiation programs [10].

NSun4 functions in mitochondria where it methylates a single cytosine (C911) of the mtDNA encoded 12S rRNA [26]. In contrast to deletion of NSun2, germline deletion of NSun4 is lethal and embryos at E8.5 are severely growth retarded and lack visible discernible anatomical structures [26]. Conditional deletion of NSun4 in the heart caused cardiomyopathy and respiratory chain deficiency due to impaired assembly of mitoribosomes and inhibition of mitochondrial translation [26].

The biological functions and targeted RNA species of NSun5 are unknown, yet its yeast homolog Rcm1 has been reported to target 25S rRNA [37]. In humans the NSun5 gene is located to a genomic region deleted in patients with Williams–Beuren syndrome, a rare neurodevelopmental disorder and lack of NSun5 may contribute to the growth retardation, the myopathy or the premature aging effects reported for the syndrome [38]. Mutations in the NSUN7 gene has been linked to infertility in mice and human due to impaired sperm motility [39, 40].

NOP2 (NSun1) is nucleolar protein that binds to 60–80S pre-ribosomal particles and is mainly described for its function in regulating cell proliferation and is up-regulated in response DNA damaging agents [41, 42]. Whether NOP2 methylates ribosomal RNA has yet to be confirmed. NOP2 is located in a genomic region deleted in patients with Cri-du-chat, a syndrome that includes a high-pitched cat-like cry, mental retardation, and microcephaly [43]. The biological functions of NSun3 and NSun6 proteins are unknown.

In summary, although the precise molecular and biological functions of RNA m^5^C methyltransferases are still poorly understood some commonalities are emerging. A conspicuously high number of NSun-proteins are associated with human disease syndromes that include growth retardation and neurological deficits. This specific link to human diseases may be explained by a direct role of 5-methylcytidine in rRNA and tRNA to regulate global protein translation. Protein synthesis pathways are coupled to cell size, which may explain the small statue described for many organisms lacking RNA methyltransferases. Another commonality is that in the absence of RNA methylases, the affected organs are often brain and testis, which both have been described to be the most susceptible organs to altered protein translation rates [44, 45].

## Post-transcriptional N^6^-methyladenosine

m^6^A is thought to be the most abundant internal modification in mRNA (Figure 1c) [46]. The detection of m^6^A was long challenging because of the inert chemical reactivity of the methyl group and the fact that this modification does not change base-pairing properties or inhibit reverse transcription. Recently, two independent groups determined the occurrence of m^6^A system-wide using RNA-immunoprecipitation methods followed by next generation sequencing [47••, 48••]. m^6^A was found in more than 7000 mRNAs and over 200 long non-coding RNAs (lncRNAs), and the conserved most pronounced location of this modification was in stop codons, 3′UTRs and long internal exons in human, mouse and yeast [47••, 48••, 49]. The consensus sequence is RRm^6^ACH (R = A/G and H = A/C/U), yet additionally RNA structure or RNA binding proteins are likely to be involved in determining the methylation sites [49]. The occurrence of m^6^A-methylation is highly dynamic, and both the fraction of modified RNAs and distribution of the modification within RNAs can vary depending on cell types, tissues and stress conditions [47••, 48••, 50••].

The addition of a single methyl group to adenosines does not perturb Watson–Crick base pairing, but it weakens RNA secondary structure [51]. Thus, the molecular role of m^6^A is thought to relate to various aspects of mRNA metabolism, including mRNA expression and degradation, splicing, translational regulation and regulation of microRNA-binding [46]. Notably, with the exception of m^6^A regulating RNA-protein interactions, there is currently a considerable lack of evidence supporting other proposed functions *in vivo*. The presence of m^6^A in mRNA modulates the binding affinity to the RNA binding proteins Hu-antigen R (HUR) and YTHDF1–3, which in turn regulate the stability and cellular distribution of the bound mRNA [47••, 52, 53].

Considering the high abundance of m^6^A in a large number of mRNAs, it is not surprising that this modification has been implicated in a wide range of cellular processes, and is likely to play an essential role in development and tissue differentiation by modulating cell fate and survival, stress responses, meiosis, the circadian clock, as wells as cellular immunity [47••, 49, 52, 54, 55, 56].

## Biological roles of N^6^-methyladenosine RNA methylases

A not yet fully characterized multicomponent complex catalyzes the formation of m^6^A in mammals. The two methylases methyltransferase-like 3 (Mettl3, also known as MT-A70) and methyltransferase-like 14 (Mettl14) form the core of the complex and associate with additional regulatory factors such as WTAP (Wilm's tumour 1 associating protein) (Figure 1c) [52, 57].

The precise biological functions of m^6^A-methyltransferases are not fully understood but emerging evidence implicates a role in embryo development, gametogenesis and stem cell self-renewal. Mouse ES cells lacking Mettl3 and Mettl14 lost self-renewal capability and the decreased levels of m^6^A in mRNAs of developmental regulators correlated with binding of the mRNA stabilizer HUR, indicating that m^6^A methylation inversely correlated with mRNA stability and is needed to maintain pluripotency [52]. During embryo development expression Mettl3 is temporarily controlled, and inactivation of the plant homolog leads to cell division defects and embryo development failure [58]. In adult flies, Mettl3 expression is highest in reproductive organs and regulates gametogenesis [59].

## Biological roles of N^6^-methyladenosine RNA demethylases

Similar to DNA m^5^C-methylation, also RNA m^6^A-methylation can be reverted. Fat mass and obesity associated protein (Fto) and α-ketoglutarate-dependent dioxygenase alkB homolog 5 (AlkBH5) are demethylases that remove m^6^A from RNA (Figure 1c) [50••, 54]. Yet, the only subtle changes in the level of m^6^A in RNA after Fto or AlkBH5 over-expression indicated substrate specificity and suggests the existence of additional demethylating enzymes [54, 60].

Genome-wide association studies linked common polymorphisms in the first intron of FTO to body mass index, risk of obesity, type 2 diabetes, polycystic ovary syndrome and cardiovascular diseases [61]. Studies in Fto loss-of-function or gain-of-function mice suggest that the main mechanism by which Fto predisposes to obesity and metabolic syndrome is driven by obesity-prone behaviors such as increased food intake and preference for high caloric food [62, 63]. Consistent with these studies, Fto inactivation in mice increased methylation of mRNAs encoding components of the dopamine signaling pathway and consequently the dopaminergic reward circuitry signaling was reduced [60]. Other human neurological conditions that have been linked to genetic variations in FTO include reduced brain volume, increased cognitive decline in elderly, dementia, Alzheimer's disease, attention deficit disorder in children and depression [64].

In addition, FTO polymorphisms in intron 1 have been linked to a range of human cancers, yet a recent meta-analysis study suggested that except for pancreatic cancer the increased cancer risk is rather associated to obesity than the FTO polymorphism itself [65]. Other polymorphisms such as in intron 8 of the FTO gene has been linked to an increased risk of developing melanoma [66].

While the functional consequences of single nucleotide polymorphisms in the intronic region of FTO are still unknown, loss-of-function mutations of FTO in humans lead to an autosomal-recessive lethal syndrome of severe growth retardation, microcephaly, psychomotor delay, cardiac deficits, and multiple malformations, and at least some of these effects may be due to impaired proliferation and accelerated senescence [67]. Similarly, Fto deficiency in mice leads to postnatal lethality, growth retardation, and multiple malformations [62].

The only limited information available about AlkBH5 indicated an essential role in gametogenesis. AlkBH5 expression is highest in primary spermatocytes in the mouse testes, and its inactivation leads to testis atrophy and infertility due to failure to enter and proceed through spermatogenic differentiation [54].

In summary, it is not fully understood how m^6^A affects the fate of methylated mRNAs and lncRNAs. While some evidence suggests that m^6^A occurrence in mRNA is inversely correlated to stability [52], it remains unclear whether specific locations within a transcript dictates distinct roles in RNA processing. What does become clear however is that m^6^A deposition plays essential roles in mRNA metabolism, and both m^6^A methylases and demethylases are crucial during embryonic development and homeostasis of the central nervous, cardiovascular and reproductive systems. Furthermore, aberrant m^6^A methylation pathways are linked to a range of human diseases including infertility, obesity as well as developmental and neurological disorders.

## Conclusions and future directions

In only a couple years, our understanding about RNA methylation pathways advanced with remarkable speed and the importance of RNA methylation and its role in human diseases is now widely recognized. However, the precise molecular pathways and cellular processes regulated by these modifications are still largely unclear. Furthermore, we only described current advances on m^5^C and m^6^A methylation, but a large number of other intriguing chemical modifications exist in RNAs. Thus, our current knowledge only scratches the surface of the many roles of post-transcriptional modifications in modulating transcriptional and translational processes.

Further advances in the field will rely on the development of new system-wide strategies to first, reliably detect m^5^C in mRNA or other low abundant RNAs, second, map m^6^A at single nucleotide resolution and third, to identify other RNA modifications. To fully understand the biological roles of RNA methylation, it will be required to identify all RNA methylases, de-methylases, the regulatory pathways that control their activity and their specific RNA targets. A major goal will be to dissect the precise mechanisms how RNA modifications affect global and specific protein production. Indeed, a modest correlation between cellular mRNA and protein levels highlights the importance post-transcriptional and post-translational regulatory pathways. Ultimately, by understanding fundamental aspects of RNA modification biology we will be able to develop selective and specific small-molecule inhibitors to modulate RNA methylation levels. Such discoveries may well lead to the identification of novel therapeutic strategies to treat complex diseases including developmental and neurological disorders, obesity or cancer.

## References and recommended reading

Papers of particular interest, published within the period of review, have been highlighted as:

• of special interest
•• of outstanding interest
